# The Role of Immunomodulation in Vein Graft Remodeling and Failure

**DOI:** 10.1007/s12265-020-10001-y

**Published:** 2020-06-16

**Authors:** Fabiana Baganha, Alwin de Jong, J. Wouter Jukema, Paul H. A. Quax, Margreet R. de Vries

**Affiliations:** 1 Department of Vascular Surgery, Leiden University Medical Center, PO Box 9600, 2300 RC Leiden, The Netherlands; 2 Einthoven Laboratory for Experimental Vascular Medicine, Leiden University Medical Center, PO Box 9600, 2300 RC Leiden, The Netherlands; 3 Aberdeen Cardiovascular and Diabetes Centre, Institute of Medical Sciences, Aberdeen University, Aberdeen, UK; 4 Department of Cardiology, Leiden University Medical Center, Leiden, The Netherlands

**Keywords:** Cardiovascular diseases, Bypass graft, Vein graft failure, Innate and adaptive immunity, Vascular remodeling, CABG

## Abstract

Obstructive arterial disease is a major cause of morbidity and mortality in the developed world. Venous bypass graft surgery is one of the most frequently used revascularization strategies despite its considerable short and long time failure rate. Due to vessel wall remodeling, inflammation, intimal hyperplasia, and accelerated atherosclerosis, vein grafts may (ultimately) fail to revascularize tissues downstream to occlusive atherosclerotic lesions. In the past decades, little has changed in the prevention of vein graft failure (VGF) although new insights in the role of innate and adaptive immunity in VGF have emerged. In this review, we discuss the pathophysiological mechanisms underlying the development of VGF, emphasizing the role of immune response and associated factors related to VG remodeling and failure. Moreover, we discuss potential therapeutic options that can improve patency based on data from both preclinical studies and the latest clinical trials. This review contributes to the insights in the role of immunomodulation in vein graft failure in humans. We describe the effects of immune cells and related factors in early (thrombosis), intermediate (inward remodeling and intimal hyperplasia), and late (intimal hyperplasia and accelerated atherosclerosis) failure based on both preclinical (mouse) models and clinical data.

## Introduction

The first saphenous vein graft (VG) implantation in humans was performed by Garrett et al. in 1967, and together with the pioneering work of Favaloro et al., VG surgery became part of the standard revascularization strategies for patients with coronary and peripheral artery diseases [1, 2]. This major advance markedly improved survival and symptoms in selected patients, but vein graft failure (VGF) may occur and this has been associated with poor outcomes, and improvements have been limited over the past decades [3, 4].

Adaptation of VGs to their new arterial environment is characterized by structural vessel wall remodeling. Moderate intimal hyperplasia (IH) and adequate outward remodeling are necessary for proper arterialization and long-term graft patency. It is well known that inflammatory processes are involved in all these phases [5]. Despite the fact that some grafts stop remodeling after arterialization, other grafts progress to a clinical stenosis and may develop advanced atherosclerosis lesions. The rate of vein graft failure is highest in the first months after graft placement. Although activation of pro-thrombotic pathways is involved, technical/anatomic issues probably dominate these failure events. This results in decreased patency rates of 10% due to acute thrombosis within the first month after surgery. Next to these early technical problems, the rate of graft failure is highest in the 3–18-month timeframe, after which the hyperplastic response and/or inward remodeling seems to become less active. After 1 year, approximately 15% of VG are occluded. After several years, there seems to be a divergence in the pathobiology of coronary versus lower extremity vein graft failure. While coronary vein graft atherosclerosis is described as the likely failure mechanism in several large series [5], the importance of atherosclerosis in lower extremity vein graft failure is not well established. By 10 years after surgery, only 60% of VG are still patent and only 50% of patent VG are free of significant stenosis, pointing out that VGF is a serious clinical problem [6–8]. Therefore, VGF limits the clinical success of coronary bypass grafting in terms of symptoms and mortality.

In this review, we discuss the pathophysiological mechanisms underlying the development of VGF, emphasizing the role of immune response and associated factors related to VG remodeling and failure. Moreover, we discuss potential therapeutic options that can improve patency based on data from both preclinical studies and the latest clinical trials.

## Mechanisms of Vein Graft Failure

VGF results from complex pathophysiological processes that lead to a partial or complete occlusion of the graft. The progression of the VGF over time involves several distinct phases and vessel wall remodeling and inflammation are central processes throughout all of them.

### Early Vascular Damage

Pre-existing quality of the venous conduit (i.e., medial hypertrophy and IH), surgical handling during harvesting, and grafting of the venous segment are all factors involved in the first stages of vessel wall remodeling [9].

Harvesting of the venous segment damages the vasa vasorum and adventitia, compromises blood supply, and thus promotes ischemia and hypoxia in the vessel wall [10]. This hypoxic state can lead to the formation of reactive oxygen radicals that damage endothelial cells (ECs) and vascular smooth muscle cells (VSMCs) [11, 12].

Usually, a high-pressure technique is used to check for leakage of ligated side-branches and reverse spams, leading to distension of the vessel and further damage of the endothelium [12, 13]. Grafting of the venous segment into an arterial environment immediately exposes the vein to an intense arterial stretch force, which further enhances the distension injury and decreases wall shear stress [14, 15]. This change in shear stress declines the production of growth inhibitors that protect the vascular wall from vasoactive substances derived from platelets—promoting thrombosis [16]. Moreover, reduced shear stress increases the production of different mitogens that promote VSMC proliferation—leading to IH [17]. Distension of the graft upregulates the expression of endothelial adhesion molecules (ICAM-1, VCAM-1, PECAM, P-Selectin) and inflammatory markers (interleukin (IL)-1, MCP-1, and TNFα via the activation of the NF-κB pathway), triggering the influx of immune cells—ultimately promoting atherosclerosis [18, 19].

### Thrombosis

Early VGF, usually defined as within hours to 1 month after grafting, is mostly due to acute thrombosis, secondary to endothelial injury and activation during VG surgery [20]. Damage of the endothelium exposes the subendothelial matrix and decreases the production of growth-inhibiting factors such as NO, heparan sulfate, and prostacyclin, creating an attractive environment for the adherence and aggregation of platelets [21]. Activated platelets secrete several pro-thrombotic substances such as tissue factor, platelet-derived growth factor (PDGF), thrombin, and plasminogen activator inhibitor-1, which initiate the coagulation cascade and fibrin deposition [22]. These processes are tightly regulated by the thrombogenic and fibrinolytic pathways, which also have important roles in the onset of IH [23]. Moreover, platelets also secrete pro-inflammatory cytokines such as MCP-1, IL-1, and IL-6, promoting leukocyte adhesion and vascular wall infiltration [24]. These interactions between activated endothelial cells and circulating platelets and leukocytes initiate an inflammatory and thrombotic cascade that can ultimately lead to thrombus formation and acute graft thrombosis [25].

### Intimal Hyperplasia

Intermediate VGF, usually defined as the period from 1 to 12 months post-surgery, is mainly caused by inward remodeling and IH [26].

**Fig. 1:**
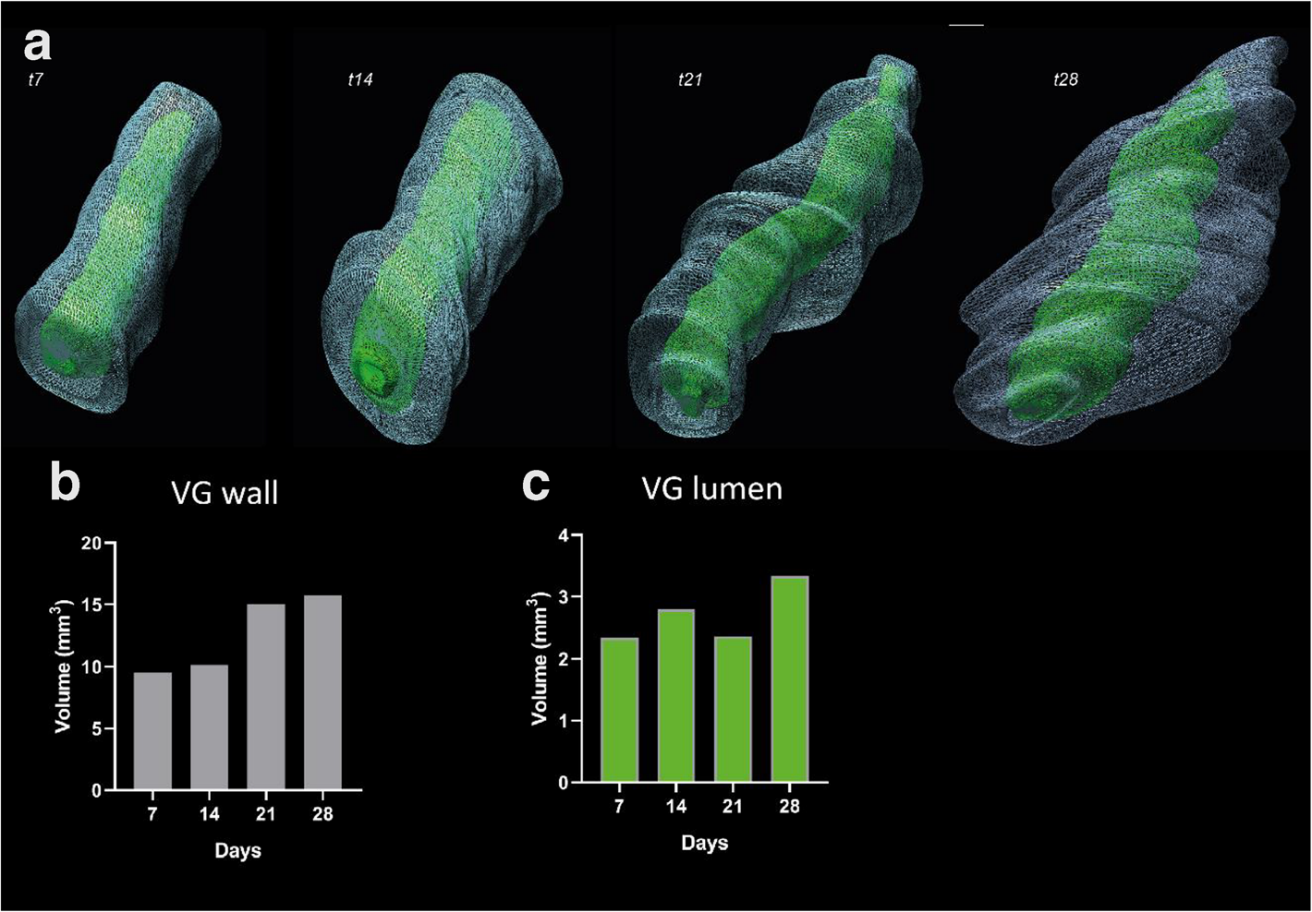
Vascular remodeling over time. Ultrasound visualization and 3D reconstruction of vein grafts (VG) in mice were obtained at 7, 14, 21, and 28 days after engraftment (A). The lumen shown in green and the VG wall in gray. An increase in VG wall volume in mm^3^ was observed (B) while the lumen volume remained comparable over time (C)

Distension under arterial pressure increases the vein diameter, compensating for the pathological lumen loss (Fig. 1). However, instead of outward remodeling, pathological IH and lumen loss can lead to inward remodeling [27].

IH starts as an adaptive response to the local arterial blood pressure and results from migration and proliferation of VSMCs from the media into the intima layer. Distension of the venous segment and endothelium damage promote an environment rich in growth factors such as TGF-β, VEGF, βFGF, and PDGF that not only activate proteases (MMPs, plasmin, cathepsins) that degrade the ECM but also stimulate uncontrolled proliferation and migration of VSMCs [28–30]. As VSMCs migrate from the media to the intima, they change their phenotype from a quiescent contractile to a proliferative synthetic state [26]. Also, adventitial fibroblasts can contribute to IH formation [31]. Veins do not contain substantial elastic laminae, and consequently, these highly proliferative fibroblasts can easily migrate to the intima. MMPs degrade components of the ECM (such as collagen) and their inhibition is associated with decreased intimal thickening [32, 33]. Overexpression of tissue inhibitor of MMPs (TIMP) inhibits MMP activity, thereby reducing VSMC migration and proliferation [34–36]. Abrogation of TGFβ signaling, which is known to enhance ECM deposition, was shown to decrease IH and increase MMP expression [37]. Plasmin, which is formed from plasminogen by plasminogen activators, can also cleave components of the ECM like laminin and fibronectin, further enhancing VSMC migration, matrix remodeling, and fibrinolysis [38]. In fact, hybrid proteins containing the amino-terminal fragment of urokinase plasminogen activator linked to a trypsin inhibitor (potent inhibitor of MMP and plasmin activity) and/or linked to TIMP decrease IH in human saphenous vein cultures and decrease IH in murine VG [39–41]. Moreover, ECM degradation products can act as endogenous ligands for TLRs, which trigger the NF-κB pathway inducing both innate and adaptive immune responses, accelerating intimal thickening and VGF [42, 43]. Vein graft remodeling is a multifactorial process in which different factors are involved. Especially microRNAs can play an interesting role in this process. MicroRNAs (miRNAs) target a multitude of genes including those that regulate gene expression in EC and VSMCs involving cell growth, differentiation, and metabolism. ECs that were mechanically stretched displayed an increase in miR-551b-5p expression [44]. The inhibition of miRNA-551b-5p reduced proliferation via inhibiting early growth response-1 (EGR-1) mRNA [45]. Mice deficient in EGR-1 showed an increased VG lumen diameter with a reduced expression of ICAM-1 [44]. miRNA-21 is upregulated in the vascular wall after injury and is able to regulate VSMC proliferation and phenotype transformation [46]. Adenovirus-mediated miR-21 sponge gene therapy not only reduced vein graft IH and suppressed VSMC proliferation but also reduced systemic effects in rats [47, 48].

In patients, miRNAs present in exosomes increased in the plasma early after coronary bypass grafting, These cardiac-derived miRNA laden exosomes could act as reporters of the myocardial injury after CABG because these miRNAs correlate with cardiac troponin-I [49].

Extensive regulation of miRNAs is observed in the vasculature as well as vein graft remodeling but needs further detailed investigation on their mechanism on VGF [50].

### Atherosclerosis and Plaque Rupture

Accelerated atherosclerosis and subsequent plaque rupture are the main causes of late VGF, and atheromatous plaques can be seen as early as 1 year after surgery [51]. The formation of atheromatous lesions is promoted by atherosclerosis predisposing factors (such as age, smoking, hypercholesterolemia, hypertension, and hyperglycemia), by vessel damage and remodeling. Pro-inflammatory cytokines contribute to vessel remodeling by stimulating VSMC proliferation and by mediating monocyte recruitment to the intima (increasing macrophage content in the VG wall) [52]. Excessive uptake of LDL induces foam cell formation and increases cholesterol deposition and necrotic core formation [53]. These accelerated atherosclerotic lesions represent an end stage in VGF and are frequently observed from 2 years onwards VG surgery [53].

**Fig. 2:**
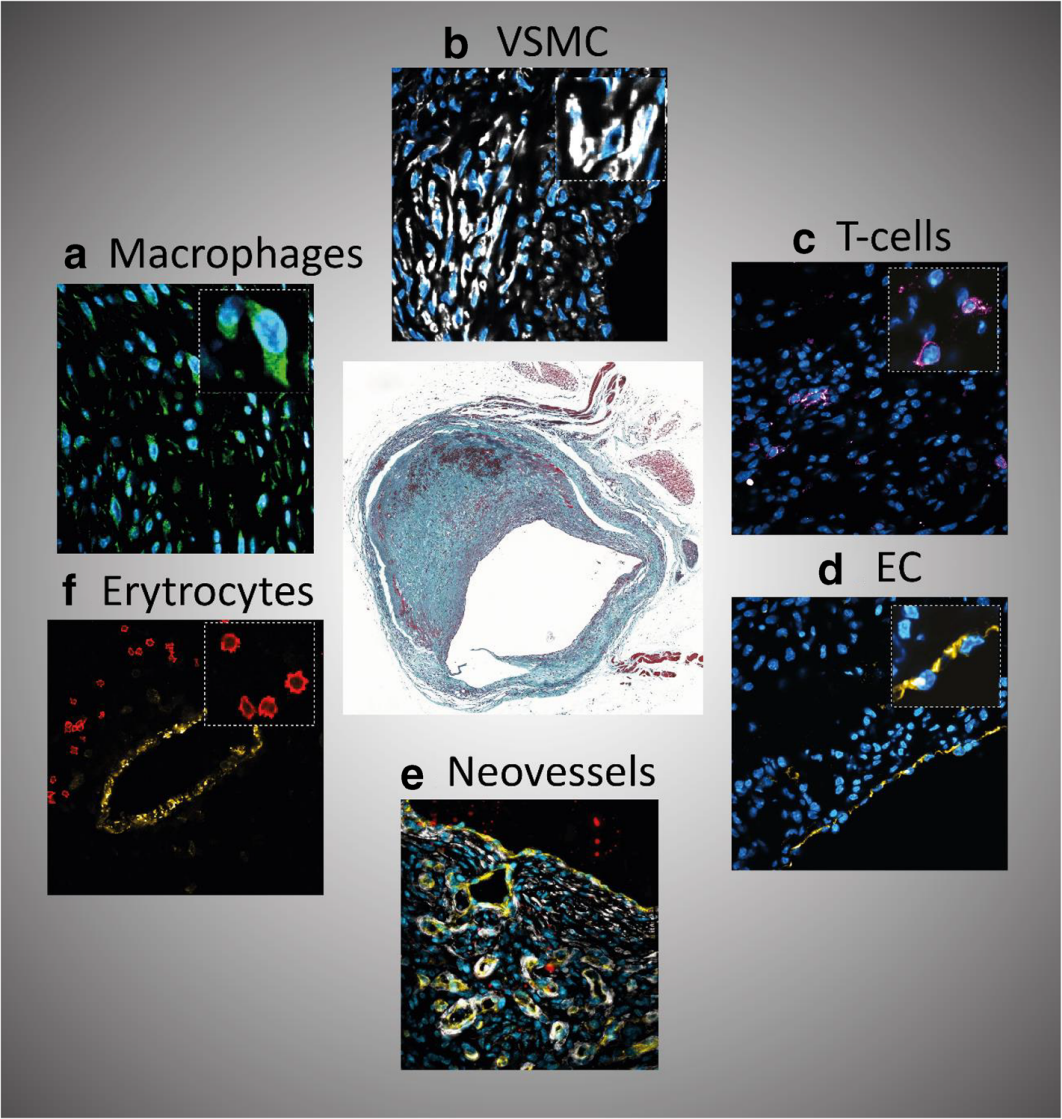
Contribution of different cells to VGF. Murine vein graft lesion (Masson’s trichrome) and (A) macrophages, MAC3 (green); (B) VSMCs, αSMA (white); (C) T cells, CD3 (pink); (D) endothelium, CD31 (yellow); (E) intraplaque angiogenesis/neovessels, CD31 (yellow); (F) intraplaque hemorrhage/erythrocytes, Ter119 (red)

VG aged more than 5 years often show necrotic core expansion through hemorrhagic events that arise from leaky neoangiogenic vessels, as shown in Fig. 2 [7, 53]. Due to the growth of the intimal layer and to the increased amount of metabolically active inflammatory cells in advance lesions, oxygen is consumed at a very high rate. ECs proliferate and migrate from the adventitia into the lesion to form neovessel-like structures and overcome the oxygen demand in the plaque. However, these neovessels are frequently immature and highly susceptible to leakage, constituting the main entrance for inflammatory cells, erythrocytes, and plasma lipids [54]. This invasion leads to a reactive, inflammatory, and apoptotic environment that profoundly affects the stability of the lesions. Neutrophils and mast cells release their granular content digesting elastin, collagen, laminin, and fibronectin, and this high proteolytic activity ultimately ends in weakening of the VG lesions including plaque erosion [55]. Furthermore, the influx and the lysis of erythrocytes drive a higher request of macrophage activity [56]. Macrophages also show a defective ability for efferocytosis. This malfunctioning increases the inflammation state and reduces cholesterol efflux contributing to necrotic core expansion and, ultimately, to plaque rupture [56].

## Immune Cells and Regulating Factors

### Toll-like Receptors and Downstream Signaling

Toll-like receptors (TLRs) are important signaling receptors within the innate as well as the adaptive immune system and are part of the primary detection system. Damaged EC as well as activated VSMC releases danger-associated molecular patterns (DAMPs) such as heat shock proteins [57]. These DAMPS are capable of activating TLRs expressed on EC, VSMC, and macrophages, although with a different pattern [43]. Upon TLR4 ligation, a downstream NF-κB-mediated pro-inflammatory response is triggered. Local application of the TLR4 ligand LPS on the VG resulted in a strong inflammatory response and an increased IH [58]. Targeting TLR4 in the murine VG model by either genetic deletion or gene silencing reduced outward remodeling and IH [57]. Interestingly, TLR2 deficiency did not result in changes in VGs [43]. Deficiency of TLR3 in the murine VG model resulted in an increase in IH, suggesting a protective role of TLR3 in VGF. Not only was the number of macrophages increased in the VG TLR3-deficient mice but also type-1 interferon expression was increased [43]. Deficiency of the TLR3 downstream factors interferon regulatory factors 3 (IRF3) or interferon regulatory factors 7 (IRF7) resulted in increased macrophage content, as well as increased IH [42]. This highlights that type-1 interferons have protective functions in VGD.

### Complement System

Beside TLR signaling, the complement system is also part of the early inflammation response/detection system in VGD. The complement system consists of a cascade of rapidly activated proteins targeting the cellular membrane in order to clear damaged cells and promote inflammation. Complement factors (C) are prominent in the human circulation and therefore present during VG surgery [59]. Inhibition of the classical complement pathway, which is initiated by C1, resulted in a reduced EC apoptosis and subsequently VG IH [60]. Exposure of the vein to the arterial pressure resulted in a transient upregulation in the C4-binding protein (C4bp) by ECs [61]. C4bp acts as a binding protein for C3a and apoptotic cells after injury, in order to reduce vascular inflammation. Also, inhibition of C3 cleavage resulted in a reduction in chemotaxis and IH in murine VGs [59]. C5a is a potent chemotaxis inducer of mast cells and monocytes. Local application of C5a on the VG resulted in an increase in mast cell presence and IH, but also and more importantly, lesion destabilization [62, 63]. Strategies in order to modulate the VG remodeling response via complement may have therapeutic benefits since mortality in CABG patients was reduced after targeting C5 by pexelizumab [64].

### Granulocytes

The VG in early remodeling is targeted by an acute inflammatory response involving granular cells such as mast cells and neutrophils. Mast cells release their histamine- and tryptase-containing granules upon activation by C5a, TNFα, IL-1, or IgE [63]. VG in mice deficient in mast cells not only showed a reduction in IH but also a general reduction in vascular inflammation [63, 65]. Neutrophils are short-lived cells and are considered early-responding cells. Neutrophils are recruited to the site of injury following signals such as C5a, IL-8, and leukotrienes. Early EC activation and damage, e.g., after the distention of the vein during graft handling and surgery, resulted in an increase in L-selectin expression and adhesion of neutrophils to ECs [66]. The involvement of neutrophils in the inflammatory response during early VG remodeling is highlighted by reduced neutrophil transmigration and reduced IH in VG in mice that received a protein-restricted diet [67].

### Monocytes

Beside granulocytes, monocytes are one of the first cells that arrive at sites of vascular injury and attach to the VG endothelium [68, 69]. Variability in the local inflammatory state could be a critical modulating factor determining the patency of VGs. Transcriptome analysis of circulating monocytes isolated from 48 patients that underwent infrainguinal venous bypass grafting resulted in three differentially expressed gene clusters. The expression of *STAT3* or *MYD88* predicted a clinically significant stenosis or thrombosis of the VG within the following year [70]. In these clusters of genes, DICER1 (a regulator gene of RNA silencing via miRNAs) was also identified [70]. Regulation of miRNAs is observed in remodeling and VGF, but needs further detailed investigation [50].

Macrophages represent a vast majority of vascular inflammatory cells contributing to VGF [71]. The expression of NOTCH delta-like ligand-4 (DII4) was abundant in failed human saphenous VGs, while control veins contained little expression of DlI4 [72]. Activation of NOTCH signaling in macrophages present in IH by DII4 contributed to the development of VGF via IL-1β, TNFα, PDGF, and impediment of immunosuppressive macrophage differentiation [73, 74]. Targeting macrophages via blockade of NOTCH and DIl4 interaction or siRNA-NOTCH present in nanoparticles resulted in reduced IH and macrophage presence [75]. Delivery of siRNA via lipid nanoparticles to target NOTCH signaling in macrophages could become an approach to reduce VG lesion development via reducing the NOTCH signaling pathway.

### T Cells

Part of the adaptive immune system are lymphocytes such as CD4^+^ and CD8^+^ T cells. CD8^+^ T cells mediate cytotoxic effects while CD4^+^ T cells modulate the immune response [76]. CD4^+^ and CD8^+^ T cells are both present and activated in VGs. Interestingly, an increased amount of CD8^+^ T cells compared with CD4^+^ T cells was observed [71, 77]. An increase in occlusions of VGs was observed when CD8^+^ T cells were depleted in vivo [71]. This highlights the protective role of CD8^+^ T cells against VGF. However, T cells are diverse and differ in effector functions that are dictated by the T cell surrounding tissue [76]. Both anti-atherogenic and pro-atherogenic effects have been demonstrated due to the diversity in effector functions within different T cell subsets. The anti-atherogenic CD8^+^ T cells were found in close proximity to caspase-3 positive cells, suggesting a cytotoxic role to control VSMC presence and function [77]. Not only T cells were involved in VG remodeling but also B cells, NK cells, and NKT cells were identified in the vascular wall of VG [71, 78].

### Antigen-Presenting Cells

Antigen-presenting cells bridge between the innate and adaptive immune system. Dendritic cells (DCs) are key antigen-presenting cells and have been shown to locate in the vessel wall. Saphenous VG contained more DCs compared with control saphenous veins [79]. These DC sense cellular debris modified metabolites and microbial infections via TLRs. The costimulatory molecule CD28 is predominantly expressed by naïve T cells and engages with CD80/86 presented by DC. This costimulatory interaction lowers the threshold for activation while the co-inhibitory molecule CTLA-4 increases the threshold for T cell activation in vascular remodeling [80]. VG from mice deficient in the costimulatory molecule CD70, CD80/86, or both showed comparable VG lumen sizes compared with control mice VG [71]. This indicated that the protective effect of CD8^+^ T cells is independent of the costimulatory molecule expression. Beside DC, ECs and VSMC are also able to activate CD4^+^ T cells and CD8^+^ T cells [81].

### Cytokines

Vascular damage during the early phase after grafting induces the release of cytokines (including chemokines, interleukins) that propagate the inflammatory response. Treatment of vein-grafted mice with the glucocorticoid dexamethasone resulted in reduced VG lesion area, as a result of reduced TNFα and, MCP-1 expression [82]. Interestingly short-term exposure to dexamethasone resulted in comparable effects as observed in long-term exposure [82].

Activation of NF-κB-mediated genes in the damaged vessel wall results in increased expression of pro-inflammatory cytokines, i.e., IL-1, MCP-1, TNFα, and TGF-β. IL-1 is involved in the initiation of adhesion molecule expression, growth factor, and cytokine release by EC and VSMC, which alters vascular function in VG remodeling [83].

In vitro, TNFα stimulates VSMC migration, proliferation, and the upregulation of adhesion molecules by EC. The response to TNFα is mediated through two receptors, P55 and P75. Both receptors are co-expressed but are differentially regulated [84, 85]. Targeting TNFα to reduce VGF showed opposing effects involving IH, wall remodeling, and influx of immune cells depending on the activated TNFα receptor.

MCP-1 (CCL2) release mediates the influx of immune cells in the VG, especially monocytes. MCP-1 recruits monocytes, memory T cells, and DC to the vascular wall via binding to the MCP-1 receptor CCR2 [86]. In vitro, gene transfer blockade of CCR2 resulted in a reduced proliferation of VSMC, and subsequently a reduction of IH in vivo without affecting cellular composition of the lesions [87, 88].

## Treatment and Therapeutic Approaches in VGF

Treatment strategies for VGF consists of thrombectomy, repeated bypass graft surgery, balloon angioplasty with or without stenting, and/or pharmacological therapies [89, 90]. The most appropriate treatment depends on the severity of symptoms, the presence and extent of ischemia, and the relative benefits and risks involved (patient’s general condition and presence of patent arterial grafts).

Antiplatelet therapy is recommended by the current guidelines, either pre- or pro-operatively, for patients undergoing VG surgery, directly aiming to address early VGF owing to acute thrombosis. A study with 25,728 patients undergoing CABG surgery showed a significant reduction in (early) VG occlusion with the use of dual antiplatelet therapy [91]. Additionally, in the DACAB trial, patients who received dual antiplatelet therapy showed a significant higher VG patency compared with patients who received mono antiplatelet therapy [92]. However, the observed higher incidence of major bleeding episodes indicates a need for risk−benefit assessment before prescription.

Statins are another mainstay as a lipid-lowering therapy in VGD patients [93]. Elevated levels of LDL are associated with IH and atherosclerotic plaque formation. High-intensity statin therapy is recommended to be administered to all patients undergoing VG surgery both before and early after surgery [93]. Non-lipid-related “pleiotropic” properties of statins might contribute to their beneficial effects that include improving EC function, increasing eNOS, and antioxidant activity [93].

Although numerous experimental studies have study gene therapy in the development of VGF, so far, only edifoligide has been assessed in the context of CABG surgery in the PREVENT series of randomized clinical trials [94]. Edifoligide is an oligonucleotide decoy that binds to and inhibits E2F transcription factors and, therefore, might prevent IH and VGF. In the PREVENT I, edifoligide treatment not only was shown to be safe and feasible but also functional [95]. Despite these initial promising results, the phase III PREVENT III and IV studies showed no differences in VGF prevention after CABG surgery between placebo and edifoligide group [96, 97]. Another promising gene therapy is the adenoviral (Ad) delivery of TIMP-1, TIMP-2, or TIMP-3 prior to grafting. Initial studies showed that ex vivo administration of Ad-TIMP-1 or Ad-TIMP-2 or Ad-TIMP-3 to human saphenous veins results in a significant inhibition of IH [34, 35]. Moreover, in short- and long-term studies, Ad-TIMP-3 delivery showed to induce VSMC apoptosis and attenuate intimal thickening in pig saphenous VGs, underlining a promise as a therapeutic approach [34, 35]. Currently, a phase-I clinical trial using an Ad-TIMP-3 ex vivo is planned at Glasgow Cardiovascular Research Center [98].

Pexelizumab, an antibody against the C5 complement, has been tested in patients undergoing VG surgery in the PRIMO-CABG trials [64]. While the PRIMO-CABG I-trial showed a reduction in death 30 days after surgery, the PRIMO-CABG II-trial was not that promising [99]. However, combined analysis of the PRIMO-CABG I and II trials showed a significantly reduction (by 2.4%) in mortality. Moreover, this observation persisted throughout the 180-day follow-up period (3.3%) [64].

A new target to prevent VG failure is phosphorylcholine (PC). PC is one of the main epitopes of oxLDL and plays a central role on its atherogenic and pro-inflammatory effects. PC epitopes can be cleared by natural IgM antibodies produced by B cells, controlling oxidative stress and inflammation. In a large human cohort, low levels of these natural antibodies were associated with a significantly increased risk of stroke, myocardial infarction, and VGF [100]. Passive immunization with anti-PC antibodies has shown to prevent VG atherosclerosis in a hypercholesterolemic murine model [101].

### Alternatives for Vein Grafting: Tissue-Engineered Grafts

Bypass surgery can be performed with different vessels, both of arterial and venous origin [5], in which the saphenous vein is most commonly used as conduit. Alternatively, grafts from prosthetic materials such as PTFE or Dacron can be used for engraftment. Despite the fact that the prosthetic engraftment of large vessels proves to be effective, the use of smaller diameter vessels is complicated by thrombotic occlusions [102]. An interesting new alternative could be the use of tissue-engineered blood vessels (TEBV) as grafts. Several variants of TEBV are described, usually based on the use of a scaffold to which vascular (precursor) cells are attracted to or seeded on [103–105].

Nanofiber vascular grafts have the potential for functional remodeling and long-term patency favoring pediatric patients. The nanofiber scaffold degrades over time allowing the induction of vascular neotissue to form vascular tissue with growing potential to form functional vessels [105]. The degradation rate orchestrates the cell infiltration and subsequently remodeling. This delicate balance between nanofiber degradation and neovessel tissue is different between species and requires optimization for the enhancement of translational capacity [103]. An alternative can be the in situ TEBV, where fibroblast and progenitor vascular cells form a vascular-like tube around a solid scaffold that can be used as a conduit for (arterio) venous grafting [104]. Together, this highlights that TEBVs may serve as arterial bypass grafts and represent a potential solution for future vascular surgery but still require optimization before large-scale clinical application is to be expected.

### Limitations

One essential limitation of the current review is that most of the pathophysiological studies are based on experimental data obtained from mouse VD studies. VGD in patients develops over years whereas the timeframe of murine VGD development is weeks. The morphological and pathological compositions of human and murine VG show similarities in the presence of calcifications, neovessels, and foam cells followed by necrotic core development. Especially the angiogenic neovessels, intraplaque hemorrhage, and necrotic cores are linked to late-phase human VGD. However, the sheer size differences between murine and human grafts may have some impact on the pathophysiology. When working with hypercholesterolemic mice, the situation observed in patient with hypercholesterolemia can be mimicked.

But the major limitation of a review on the pathophysiology of vein graft failure and the role of immunomodulation in this process is that, although not yet described, the quality of the surgical intervention is of eminent importance and can vary a lot, with all the consequences on long-term vein graft patency.

## Conclusions and Perspectives

Preclinical studies have demonstrated the role of the immune system in VG remodeling and IH and in unstable atherosclerotic lesions in VG, the main causes of VGF. Therefore, therapeutic modulation of the immune system may represent a step forward in the prevention of VGF but further research is needed.
